# Usefulness of multimodal MR imaging in the differential diagnosis of HaNDL and acute ischemic stroke

**DOI:** 10.1186/1471-2377-10-120

**Published:** 2010-12-02

**Authors:** Tomas Segura, Francisco Hernandez-Fernandez, Pedro Sanchez-Ayaso, Elena Lozano, Lorenzo Abad

**Affiliations:** 1 Department of Neurology. Hospital Universitario de Albacete. C/ Hermanos Falcó. Albacete 02006, Spain; 2 Department of Neuroradiology. Hospital Universitario de Albacete

## Abstract

**Background:**

Syndrome of transient Headache and Neurological Deficits with cerebrospinal fluid Lymphocitosis (HaNDL) is a rare disease which can present with focal neurological deficits and mimic stroke. A neurologist-on-duty faced with a HaNDL patient in the first hours might erroneously decide to use thrombolytic drugs, a non-innocuous treatment which has no therapeutic effect on this syndrome.

**Case Presentation:**

We present a case where neuroimaging, together with the clinical picture, led to a presumed diagnosis of HaNDL avoiding intravenous thrombolysis.

**Conclusions:**

This report shows the usefulness of multimodal MR imaging in achieving early diagnosis during an acute neurological attack of HaNDL. Our experience, along with that of others, demonstrates that neuroimaging tests reveal the presence of cerebral hypoperfusion in HaNDL syndrome

## Background

Syndrome of transient Headache and Neurological Deficits with cerebrospinal fluid Lymphocitosis (HaNDL, ICHD-II 7.8), previously termed as pseudomigraine with lymphocytic pleocytosis, is a rare disease that was first described by Bartleson et al in 1981 (1). The International Classification of Headache Disorders diagnostic criteria for HaNDL are: (A) episodes of moderate or severe headache lasting hours; (B) CSF pleocytosis with lymphocytic predominance and normal neuroimaging, CSF culture and other tests for aetiology; (C) episodes of headache are accompanied by transient neurological deficits; and (D) episodes of headache and neurological deficits recur over <3 months (2). Thereafter, HaNDL is an exclusion diagnosis that mainly depends upon the experience and awareness of the physician (3). Moreover, given that it is an acute or subacute disease which presents with focal neurological deficits, it may mimic stroke. With the progressive implementation of regional systems for rapid identification and transport of acute stroke patients, or "stroke code" programs, transferring a HaNDL patient to hospital in order to consider thrombolysis treatment is now more feasible. Since thrombolytic treatments are not innocuous, it is very important for neurologists to be aware of HaNDL syndrome and, if possible, to have at their disposal fast diagnostic tools to differentiate it from acute brain ischemia.

We present a case in which multimodal MR imaging (diffusion-weighted imaging/perfusion-weighted imaging/MR angiography) helped in early diagnosis of HaNDL, avoiding intravenous thrombolysis.

## Case Presentation

A 38-year-old woman with no relevant medical history was brought into our emergency department for urgent neurological evaluation. Her relatives described a sudden language disturbance which had started less than three hours earlier. They also explained that throughout the previous day she had complained of gradually worsening holocranial headache and had suffered vomiting. There was no prior history of migraine or toxic abuse. On examination, the patient was slightly agitated and showed global aphasia without any other neurological deficit. The National Institutes of Health Stroke Scale (NIHSS) scored 6 points, all of them from the area of language.

**Figure 1:**
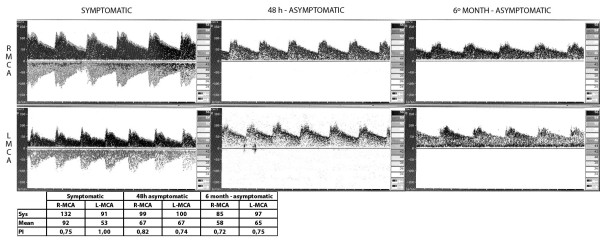
**TCD showing a TIBI 3-like pattern during the symptomatic period**. Subsequent normalization at 48 hours and 6 month, asymptomatic periods. VSys: Systolic velocity; VMean: Mean velocity PI: Pulsatility index (Gosling).

**Figure 2:**
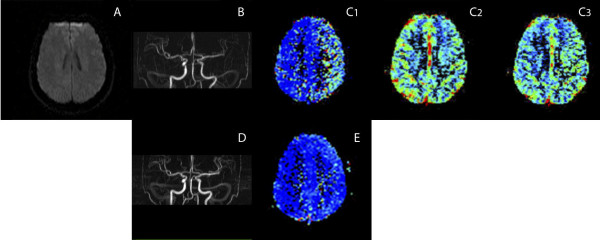
**Multimodal brain MRi; axial images**: (A) Diffusion-weighted scans demonstrating no abnormalities; (B) MR angiogram (3DPC) of the circle of Willis showing narrowing of the left ICA and branches of the MCA and PCA; (C_1_). Prolonged mean transit time (red color denotes prolongation) in the left hemisphere with respect to the right one, with (C2) mild reduction in cerebral blood flow, and (C_3_) mild increase of cerebral blood volume. Three months later (D) MR angiogram was normal. The maps of CVB and CBF were also normal and (E) the map of mean transit time had almost normalized.

A non-contrast cerebral CT scan, performed 185 minutes after the aphasia onset, was normal, revealing no signs of brain ischemia or hemorrhage. A transcranial Doppler study (TCD), immediately carried out after CT scan, showed velocity asymmetry between both middle cerebral arteries (MCA) compatible with a left MCA TIBI 3 pattern. An embolic stroke was suspected and intravenous thrombolysis was proposed. As the time elapsed since the aphasia onset was now over 3 hours, a multimodal brain MR study was made following our centerÂ´s protocol. Although the images revealed extensive delayed perfusion in the whole left hemisphere, including the territory of anterior (ACA), MCA and posterior (PCA) cerebral arteries, there were no altered diffusion-weighted images (Figure 1). In the MR-angiography the diameter of left MCA and its main branches was reduced with respect to the contralateral artery (Figure 2). There was no fetal origin of left PCA or flow alteration in terminal internal carotid artery. The existence of a complete hemispheric perfusion/diffusion mismatch, together with the clinical picture including the presence of headache, led to a presumed diagnosis of HaNDL (ICHD-II 7.8). A lumbar puncture was performed with a CSF opening pressure of 18 cmH_2_0, 80 cells/mm^3 ^(95% lymphocytes), 0.50 g/L proteins and 82 mg/dL glucose. These results were congruent with the suspected diagnosis. Invasive procedures or treatments were avoided, and the patient was admitted to a follow-up of her evolution. Eighteen hours later her symptoms had improved, and 2 days after admission her NIHSS scored 0. A new TCD study showed symmetric MCAs velocities.

CSF stains and cultures were normal. Additional tests performed (CBC, ESR, biochemistry, thyroid hormones, vitamin B12 and cold agglutinin) showed normal values. Borreliosis, syphilis, brucellosis, mycoplasma, hepatitis B, C and HIV serologies were negative. Studies of autoimmunity were carried out (ANA, anti-DNA, anti-Ro, anti-LA, anti-RNP, p-ANCA, c-ANCA, IgG and IgM cardiolipin), all yielding negative results. Given the presence of CSF pleocytosis and the patient's clinical evolution, we did not consider a high probability of reversible cerebral vasoconstriction syndrome or primary CNS vasculitis. Moreover, since it has been described that cerebral angiography could worsen neurological deficits in HaNDL, conventional arteriography was not performed.

After 30 days the patient suffered another episode. She came back to the emergency department with worsening headache, which had started 10 hours earlier, accompanied by vomiting without fever or other neurological deficits. The patient recalled the intensity and characteristics of headache as identical to that of the previous event. CT without contrast was again performed, showing no ischemic or hemorrhagic lesions (images not shown). A new TCD study displayed no abnormalities in mean blood flow velocity (MFV) of intracranial arteries. Lumbar puncture was not done because there was no neurological deficit and the suspicion of HaNDL recurrence was high. The episode was resolved with 50 mg of intravenous dexketoprofen. There were no further recurrences. We repeated TCD and multimodal-MRi four months later. These explorations were completely normal, including FLAIR images, with symmetric findings in both hemispheres (Figures 1 and 2).

## Discussion and Conclusions

In HaNDL syndrome, episodes of headache are accompanied by transient neurological deficits. The neurological manifestations, involving either cerebral hemisphere and/or the brainstem/cerebellum, are most commonly sensory symptoms or aphasia. In the latter case, a fast diagnosis of HaNDL is even more difficult due to the patient's inability to fully explain symptoms. In both hyperacute stroke and HaNDL, routine CT scans are normal and a neurologist-on-duty faced with a HaNDL patient in the first hours may thus erroneusly decide to use thrombolytic drugs, a non-innocuous treatment. Moreover, transcranial Doppler, a useful help for neurologists who treat acute stroke patients, may increase confusion in this scenario; like in our case, it can show focal disturbances (4), which are liable to be misinterpreted as a TIBI pattern consistent with distal occlusion and embolism. Other tests, such as single photon emission CT (SPECT) (5), could also demonstrate focal hypoperfusion in HaNDL syndrome, but in the first hours after the onset of symptoms this information is not sufficient to allow differential diagnosis of HaNDL and stroke. Apart from that, SPECT is not usually available to study acute stroke patients in the emergency room. Recently, CT perfusion has been described as a valid test to differentiate stroke from HaNDL in a single case report (6). Other recent reports also show global hemispherical hypoperfusion in HaNDL syndrome (7,8). In these previous reports, as well as in ours, there were no areas of brain infarction and the perfusion techniques give evidence of a decreased and delayed perfusion pattern in one hemisphere, although the angiograms were normal. These findings were not compatible with an embolic stroke, and patients were therefore not thrombolysed. In our case, multimodal MR imaging was used to assess a possible perfusion/diffusion mismatch in a suspected stroke patient who had more than 3 hours of evolution. The results of the test suggested a stroke mimic, and the patient's medical history, together with the awareness of this special illness, led to a presumptive diagnosis of HaNDL. Both the complete negative etiological study and the evolution confirmed diagnosis.

At least 3% of all patients treated with thrombolysis for acute stroke actually suffer a neurovascular mimic condition (9). Published experience show that treating stroke mimics with intravenous alteplase does not involve a high risk of cerebral hemorrhage (10). However, this fact can not ignore that in any medical case, clinical suspicion should be the guiding principle of action. Nowadays, many tools may improve hyperacute stroke diagnosis and reduce mimic treatment rates. If available, neurologist should use them. In case of doubt or if additional tools are not available, then thrombolysis should be seriously considered.

Although HaNDL is a rare disease, the neurologist should be aware of it, mainly in the emergency room. In a suspected stroke patient, the existence of a complete hemispheric perfusion/diffusion mismatch with normal diffusion imaging, together with the clinical picture -including the presence of accompanying headache and vomiting (11)-, may lead to a presumed diagnosis of HaNDL.

In spite of the ICHD-II diagnostic criteria, which state that neuroimaging findings are normal in HaNDL, our experience, along with that of others (5-8), demonstrates that neuroimaging tests reveal the presence of cerebral hypoperfusion in this syndrome. It is proposed that this new information be added in a future revision of the International Classification of Headache Disorders.

## Competing interests

The authors declare that they have no competing interests.

## Authors' contributions

TS and FHF cared for the patient in the emergency department, participated in the design of the case-report, and drafted the manuscript. EL and LA performed and interpreted the MRi studies, and participated in the design of the case-report. PSA treated the patient in his office, was involved in the design of the case-report, and drafted the manuscript. All authors contributed to the critical review and approval of the final draft.

## Pre-publication history

The pre-publication history for this paper can be accessed here:

http://www.biomedcentral.com/1471-2377/10/120/prepub
